# Supplementary material for: Taxonomic distinctness and diversity patterns of a polychaete (Annelida) community on the continental shelf of the Southern Gulf of Mexico
Source: PLoS One. 2024 May 8;19(5):e0303250. doi: 10.1371/journal.pone.0303250 (PMC11078371; doi:10.1371/journal.pone.0303250)
Supplement: Attachment — Submitted filename: PONE-D-23-19360_reviewer22.pdf [file pone.0303250.s005.pdf]

PLOS ONE

Taxonomic distinctness and diversity patterns of a polychaete (Annelida) community in the continental shelf of the Southern Gulf of Mexico

--Manuscript Draft--

**Manuscript Number:**	PONE-D-23-19360
**Article Type:**	Research Article
**Full Title:**	Taxonomic distinctness and diversity patterns of a polychaete (Annelida) community in the continental shelf of the Southern Gulf of Mexico
**Short Title:**	Taxonomic distinctness patterns of a polychaetes in the continental shelf of the Southern Gulf of Mexico
**Corresponding Author:**	Benjamín Quiroz-Martínez<br>Instituto de Ciencias del Mar y Limnología<br>Mexico City, MEXICO
**Keywords:**	Annelid polychaetes, Gulf of Mexico, Taxonomic Distinctness, Distance-based Moran's eigenvector maps
**Abstract:**	The spatial patterns of taxonomic diversity of annelid polychaete species from the continental shelf in the Southern Gulf of Mexico were examined in this study. We used taxonomic distinctness and its spatial variations to explore the diversity patterns and how they change between Southern Gulf of Mexico regions. In addition, using taxonomic distinctness as a dissimilarity measure and Ward's Clustering, we characterised three distinct faunal assemblages. We also investigated patterns of richness, taxonomic distinctness, and distance decay of similarity between sampling stations as a ß-diversity measure. Finally, we examined the spatial relationships between polychaete assemblages and environmental variables to test the relative importance of spatial and environmental components in annelid polychaete community structure from the Southern Gulf of Mexico. We used a combination of eigenvector-based multivariate analyses (dbMEMs) and distance-based redundancy analysis (dbRDA) to quantify the relative importance of these explanatory variables on the spatial variations of taxonomic distinctness. The significance level of spatial and environmental components to the distribution of polychaete species showed that the combined effect of spatial processes and sediment characteristics explained a higher percentage of the variance than those parameters could alone.
**Order of Authors:**	Benjamín Quiroz-Martínez<br>Pablo Hernández-Alcántara<br>David Alberto Salas de León<br>Vivianne Solís-Weiss<br>Maria Adela Monreal-Gómez, Doctorate<br>León Felipe Álvarez Sánchez
**Additional Information:**	
**Question**	**Response**
**Financial Disclosure**<br><br>Enter a financial disclosure statement that describes the sources of funding for the work included in this submission. Review the submission guidelines for detailed requirements. View published research articles from *PLOS ONE* for specific examples.	The funders had no role in study design, data collection and analysis, decision to publish, or preparation of the manuscript.

Powered by Editorial Manager® and ProduXion Manager® from Aries Systems Corporation

This statement is required for submission and **will appear in the published article** if the submission is accepted. Please make sure it is accurate.<br><br>**Unfunded studies**<br>Enter: *The author(s) received no specific funding for this work.*<br><br>**Funded studies**<br>Enter a statement with the following details:<br>• Initials of the authors who received each award<br>• Grant numbers awarded to each author<br>• The full name of each funder<br>• URL of each funder website<br>• Did the sponsors or funders play any role in the study design, data collection and analysis, decision to publish, or preparation of the manuscript?<br>• **NO** - Include this sentence at the end of your statement: *The funders had no role in study design, data collection and analysis, decision to publish, or preparation of the manuscript.*<br>• **YES** - Specify the role(s) played.<br><br>* typeset	
**Competing Interests**<br><br>Use the instructions below to enter a competing interest statement for this submission. On behalf of all authors, disclose any competing interests that could be perceived to bias this work—acknowledging all financial support and any other relevant financial or non-financial competing interests.<br><br>This statement is required for submission and **will appear in the published article** if the submission is accepted. Please make sure it is accurate and that any funding sources listed in your Funding Information later in the submission form are also declared in your Financial Disclosure statement.<br><br>View published research articles from *PLOS ONE* for specific examples.	The authors have declared that no competing interests exist.

**NO authors have competing interests**<br><br>Enter: *The authors have declared that no competing interests exist.*<br><br>**Authors with competing interests**<br><br>Enter competing interest details beginning with this statement:<br><br>*I have read the journal's policy and the authors of this manuscript have the following competing interests: [insert competing interests here]*<br><br>* typeset	
**Ethics Statement**<br><br>Enter an ethics statement for this submission. This statement is required if the study involved:<br><br>• Human participants<br>• Human specimens or tissue<br>• Vertebrate animals or cephalopods<br>• Vertebrate embryos or tissues<br>• Field research<br><br>Write "N/A" if the submission does not require an ethics statement.<br><br>General guidance is provided below. Consult the submission guidelines for detailed instructions. **Make sure that all information entered here is included in the Methods section of the manuscript.**	Samples were collected during six oceanographic expeditions, on board the R/V “Justo Sierra” (Universidad Nacional Autónoma de México), from 1988 to 1996, as part of the institutional projects “IMCA” and “DINAMO”

**Format for specific study types**<br><br>**Human Subject Research (involving human participants and/or tissue)**<br>• Give the name of the institutional review board or ethics committee that approved the study<br>• Include the approval number and/or a statement indicating approval of this research<br>• Indicate the form of consent obtained (written/oral) or the reason that consent was not obtained (e.g. the data were analyzed anonymously)<br><br>**Animal Research (involving vertebrate animals, embryos or tissues)**<br>• Provide the name of the Institutional Animal Care and Use Committee (IACUC) or other relevant ethics board that reviewed the study protocol, and indicate whether they approved this research or granted a formal waiver of ethical approval<br>• Include an approval number if one was obtained<br>• If the study involved *non-human primates*, add *additional details* about animal welfare and steps taken to ameliorate suffering<br>• If anesthesia, euthanasia, or any kind of animal sacrifice is part of the study, include briefly which substances and/or methods were applied<br><br>**Field Research**<br><br>Include the following details if this study involves the collection of plant, animal, or other materials from a natural setting:<br>• Field permit number<br>• Name of the institution or relevant body that granted permission	
**Data Availability**<br><br>Authors are required to make all data underlying the findings described fully available, without restriction, and from the time of publication. PLOS allows rare exceptions to address legal and ethical concerns. See the PLOS Data Policy and FAQ for detailed information.	No - some restrictions will apply

Powered by Editorial Manager® and ProduXion Manager® from Aries Systems Corporation

A Data Availability Statement describing where the data can be found is required at submission. Your answers to this question constitute the Data Availability Statement and **will be published in the article**, if accepted.<br><br>**Important:** Stating 'data available on request from the author' is not sufficient. If your data are only available upon request, select 'No' for the first question and explain your exceptional situation in the text box.<br><br>Do the authors confirm that all data underlying the findings described in their manuscript are fully available without restriction?	
**Describe where the data may be found in full sentences. If you are copying our sample text, replace any instances of XXX with the appropriate details.**<br><br>• If the data are **held or will be held in a public repository**, include URLs, accession numbers or DOIs. If this information will only be available after acceptance, indicate this by ticking the box below. For example: *All XXX files are available from the XXX database (accession number(s) XXX, XXX.).*<br>• If the data are all contained **within the manuscript and/or Supporting Information files**, enter the following: *All relevant data are within the manuscript and its Supporting Information files.*<br>• If neither of these applies but you are able to provide **details of access elsewhere**, with or without limitations, please do so. For example:<br><br>*Data cannot be shared publicly because of [XXX]. Data are available from the XXX Institutional Data Access / Ethics Committee (contact via XXX) for researchers who meet the criteria for access to confidential data.*<br><br>*The data underlying the results presented in the study are available from (include the name of the third party*	Data will be posted in Obis in the future but is available from request from the author

and contact information or URL).<br>• This text is appropriate if the data are owned by a third party and authors do not have permission to share the data.<br><br>* typeset	
Additional data availability information:	

Manuscript
Click here to access/download;Manuscript;Quiroz et al Plos 2023.docx

|Taxonomic distinctness and diversity patterns of a polychaete (Annelida) community in the continental shelf of the Southern Gulf of Mexico

[1*]Benjamín Quiroz-Martínez, [1]Pablo Hernández-Alcántara, [1]David Alberto Salas-de León, [2]Vivianne Solís-Weiss, [1]María Adela Monreal-Gómez, [3]León Felipe Álvarez-Sánchez

[1]Unidad Académica de Ecología y Biodiversidad Acuática, Instituto de Ciencias del Mar y Limnología, Universidad Nacional Autónoma de México. Circuito Exterior S/N, Cd. Universitaria, Ciudad de México, 04510, México

[2]Unidad Académica de Sistemas Arrecifales, Instituto de Ciencias del Mar y Limnología, Universidad Nacional Autónoma de México. Prol. Av. Niños Héroes s/n Puerto Morelos Quintana Roo, 77580, México

[3]Unidad de informática Marina, Instituto de Ciencias del Mar y Limnología, Universidad Nacional Autónoma de México, Circuito Exterior S/N, Cd. Universitaria, Ciudad de México, 04510, México

***Corresponding author: Benjamín Quiroz-Martínez (bquirozm@cmarl.unam.mx)**

ABSTRACT

The spatial patterns of taxonomic diversity of annelid polychaete species from the continental shelf in the Southern Gulf of Mexico were examined in this study. We used taxonomic distinctness and its spatial variations to explore the diversity patterns and how they change between Southern Gulf of Mexico regions. In addition, using taxonomic distinctness as a dissimilarity measure and Ward's Clustering, we characterized three distinct faunal assemblages. We also investigated patterns of richness, taxonomic distinctness, and distance decay of similarity between sampling stations as a ß-diversity measure. Finally, we examined the spatial relationships between polychaete assemblages and environmental variables to test the relative importance of spatial and environmental components in annelid polychaete community structure from the Southern Gulf of Mexico. We used a combination of eigenvector-based multivariate analyses (dbMEMs) and distance-based redundancy analysis (dbRDA) to quantify the relative importance of these explanatory variables on the spatial variations of taxonomic distinctness. The significance level of

spatial and environmental components to the distribution of polychaete species showed that the combined effect of spatial processes and sediment characteristics explained a higher percentage of the variance than those parameters could alone.

Keywords: Annelid polychaetes, Gulf of Mexico, Taxonomic Distinctness, Distance-based Moran's eigenvector maps

Introduction

Explaining the causes of biodiversity variability at various scales and correlating these variations to changes in environmental conditions are two of ecology's primary goals (Benedetti-Cecchi et al., 2010; Economo & Keitt, 2008). To estimate how biodiversity may respond to climate change, it is essential to understand the patterns of species distribution and the ecological and evolutionary processes that lead to these patterns. Abiotic factors influence the borders of the species ranges based on their physiological preferences (Chase & Leibold, 2003). Numerous complex mechanisms have determined the distribution of species at diverse geographical and temporal scales (Leibold et al., 2004).

Over the past few years, there has been a remarkable growth in research on marine biodiversity patterns, and understanding the spatial distribution of species in soft-bottom habitats is essential to comprehend how species interact with one another and with the environment (Dauvin & Bellan-Santini, 2004) and how benthic communities and ecosystems function (Checon & Amaral, 2017; Legendre et al., 2005). Community ecology studies frequently address the connection between species composition and environmental factors (Alves et al., 2020; Giangrande & Gravina, 2015; Mikac et al., 2020; Quiroz-Martínez et al., 2021; Semprucci et al., 2019; Thrush et al., 2010). Spatially structured environmental variation plays a vital role in species distribution (Dray et al., 2012); consequently, discriminating spatial ecological structures in species composition, distribution, and diversity is a pivotal question in community ecology (Legendre, 1993). However, the spatial scale can also incorporate puzzling factors; thus, statistical models should include them as predictors or covariable parameters (Dray et al., 2006). The local scale is generally considered the spatial autocorrelation created by community dynamics, while the regional scale corresponds to the scale of the environmental drivers (Dray et al., 2012).

The spatial component in community analysis has been incorporated using a variety of methodologies (Dormann et al., 2007; Keitt et al., 2002). For example, ordination analysis (Borcard et al., 1992; Legendre, 2018) can partition the variance across different variables. This approach entails assessing environmental variables, creating a spatial coordinates matrix, and characterizing these elements' individual and combined contributions to the community structure. Due to its ability to explain the regional and local dynamics that shape communities, variation partitioning has become a crucial exploratory tool (Checon & Amaral, 2017; Gilbert & Bennett, 2010; Heino et al., 2012). Processes associated with sediment properties, temperature, salinity, dissolved oxygen, and nutrient content are among the drivers that structure the geographical distribution of biodiversity (Levin et al., 2001; Quiroz-Martínez et al., 2021; Schnurr et al., 2018).

Annelid polychaetes are among the most diverse and characteristic groups of benthic macroinvertebrates (Hutchings, 1998; Knox, 1977) and one of the richest concerning species numbers (Arvanitidis et al., 2002; Capa & Hutchings, 2021); it includes nearly 11,500 accepted nominal species and is often the dominant group in benthic macrofauna (Pamungkas et al., 2019), representing up to 60% of the species and over 70% of the abundance, mainly in soft-bottom communities (Dogan et al., 2005; Knox, 1977). They are ubiquitous in soft sediments and show significant spatial distribution patterns under the influence of environmental gradients (Gopal et al., 2020). Polychaetes can be used as "indicators" of different ecological conditions (Gambi & Giangrande, 1986; Quiroz-Martínez et al., 2012), and they can be particularly informative in assessing the health of benthic environments. Therefore, the analysis of the diversity patterns of this fauna can be crucial in understanding the whole ecosystem functioning since the biological processes that determine these patterns can reflect those of the entire ecosystem (Belan, 2003; Ergen et al., 2006; Giangrande et al., 2005; Glasby & Read, 1998; Mackie et al., 1997; Mutlu et al., 2010; Olsgard & Somerfield, 2000). In the continental shelf of the Gulf of Mexico, the polychaetes have been the focus of comprehensive research for decades as they represent a crucial group in abundance and diversity (Fauchald et al., 2009); however, most of these studies only present taxonomic lists and are often presented in a fragmented or isolated way; thus, information about their distribution patterns related to environmental factors is still scarce. A comprehensive polychaete database was therefore built using the results of several research projects examining the occurrence of this group in the continental shelf of the Southern Gulf of Mexico.

Using distance-based redundancy analysis (dbRDA) and variation partitioning, this study aims to analyze the environmental factors that influence polychaete diversity at small and intermediate scales along the continental shelf of the Southern Gulf of Mexico: 1) assessing the relative impact of spatial and environmental components on polychaete community structure; and 2) examining the spatial relationships between polychaete assemblages and environmental variables. A common approach to examining spatial ß-diversity is considering a directional turnover in community composition with distance (Distance Decay). In this framework, the similarity amongst pairs of biological communities typically decreases (decays) with increasing spatial and environmental distance.

2. Materials and Methods

2.1. Study area

The Gulf of Mexico is a marginal sea shared by three countries, Mexico, Cuba, and the United States of America. It is recognized as a large marine ecosystem (Dorr et al., 2019), where activities with significant economic importance, such as fisheries, transportation, and gas and oil extraction, constantly increase (Dorr et al., 2019). The Southern Gulf of Mexico (18° 30′22° 20′ N; 89° 41′–97° 4′ W), delimited by an imaginary line that goes from the port of Tampico, in the State of Tamaulipas to Progreso in the State of Yucatan covers approximately 46 000 km^2. Its southeastern portion (Fig. 1, upper panel) is composed of two distinct regions: The Campeche Bank, an expansive shelf characterized by a gentle slope and uneven bottoms and sandbanks, coral reefs, and native biogenic and autogenic sediments along most of the Yucatan Peninsula's coast, is to the east of the Bay of Campeche, which is a narrow shelf with its upper limit at 130 m depth and between 45 and 65 km wide (Fig. 1) (Antoine, 1972; Martin & Bouma, 1978). Three seasons characterize the southern Gulf of Mexico, the dry season from March to May, the rainy season from June to October, and the winter storms season, locally called the "Nortes" season, from November to February (López-Cabello et al., 2022). The southern Gulf of Mexico has combined influences from river run-off to the west and winter storms ("Nortes") (Fuentes-Yaco et al., 2001; Hernández-Arana et al., 2005). The study area has been classified according to the sediments' carbonate content: the "terrigenous" region to the west with less than 25% and the "carbonate" region to the east and north, with more than 75% with a transitional carbonate-terrigenous region,

between the first two, whose limits change throughout the year, with intermediate values (Carranza-Edwards et al., 1993; Hernández-Arana et al., 2003; Ortiz-Zamora et al., 2002). The terrigenous sediments are predominantly silty with variable amounts of gravel, sand, and clay, while fragments of shells, corals, and algae mainly constitute the carbonate ones. The Southern Gulf of Mexico is a complex tropical domain with intense river discharges to the west from the Grijalva-Usumacinta River system, which modifies the temperature and salinity, inducing coastal frontogenetic processes, in addition to being the primary source of terrigenous sediments (Carranza-Edwards et al., 1993; Monreal-Gómez et al., 1992). Discharges from the Grijalva-Usumacinta and San Pedro-San Pablo rivers, “Nortes”, summer rains, and intrusion of oceanic water from the Caribbean Sea are the physical driving processes on the continental shelf of the southern Gulf of Mexico (Hernández-Arana et al., 2005; Monreal-Gómez et al., 1992). The Caribbean current drives the water circulation pattern in the spring and summer, with a south-southwest flow; however, in the autumn and winter, the flow shifts to an east-northeast direction (Boicourt et al., 1998; Hernandez Arana et al., 2005; Salas-Monreal et al., 2018). As the Caribbean Current flows northward through the Yucatan Channel, it is renamed the Yucatan Current. The Caribbean Tropical Surface Water (CTSW: $T>28$ °C and $S>36.4$) and the Caribbean Subtropical Underwater (CSUW: $22.2<T<26$ °C and $36.4<S<36.7$) influence the Campeche Bank since the Yucatan current flows into the Gulf, not only contributing significantly to the Loop Current, but another branch flows westward on the Yucatan continental shelf and Campeche Bank (Aldeco-Ramírez et al., 2009).

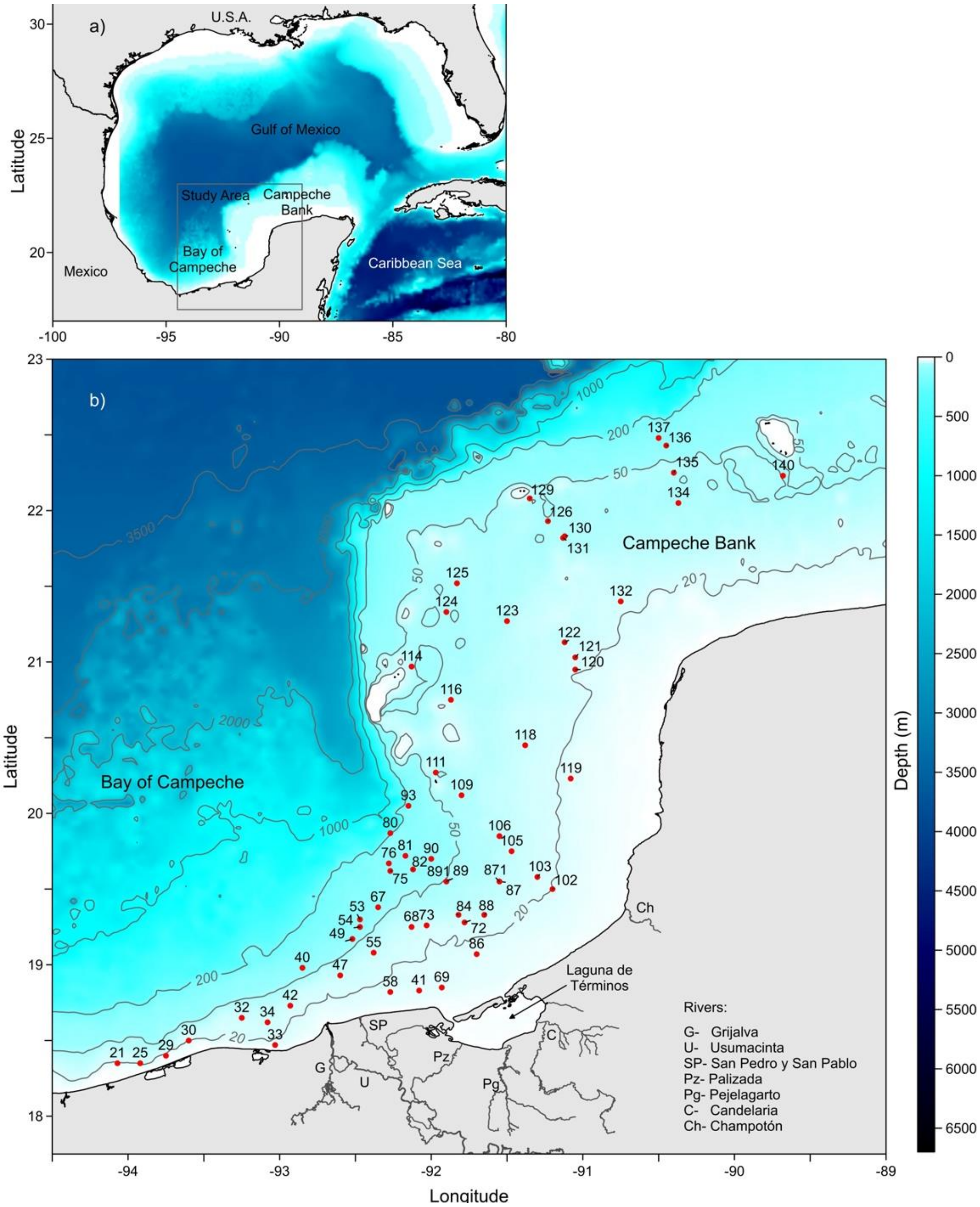

Figure 1. Sampling stations in the Southern Gulf of Mexico.

2.2. Data source

The polychaete database, constructed from extensive studies carried out on the continental shelf (< 200 m depth) of the Southern Gulf of Mexico (18° 30′–22° 20′ N; 89° 41′–97° 4′ W) was the primary source of information: the samples were collected during six oceanographic expeditions, on board the R/V "Justo Sierra" (Universidad Nacional Autónoma de México), from 1988 to 1996, as part of two institutional projects "IMCA" and "DINAMO" (Solís-Weiss et al., 1995). The

collected specimens from 61 stations were identified to species level and deposited in the "Colección Nacional de Anélidos Poliquetos" from the Instituto de Ciencias del Mar y Limnología, Universidad Nacional Autónoma de México (CNAP-ICML, UNAM; DFE.IN.061.0598). The database includes information on all identified polychaete species, the geographical position of the sampling stations (latitude and longitude), and environmental variables such as sediment composition, depth, temperature, and salinity where they were collected. In addition, the taxonomic information was carefully checked. We verified the validity of names and synonymies and omitted species whose taxonomic identification was doubtful. We used the more recent systematic reviews and the World Polychaeta Database (Read & Fauchald, 2021) accessed through the World Register of Marine Species (WORMS) database.

2.3. Statistical analyses

The information in the database was linked to the corresponding supra-generic levels for every species; their relationships to genus, family, and class were included. We then calculated two indices to compare polychaete diversity between sampling stations: 1) the Average taxonomic distinctness (Δ^+), and 2) the variation in taxonomic distinctness (Λ^+), as defined by Warwick & Clarke (2001) and Clarke & Warwick (1998).

The Average taxonomic distinctness (Δ^+) was calculated using the function:

$$\Delta^+ = \left[\Sigma\Sigma_{i<j}\omega_{ij}\right]/[S(S-1)/2] \qquad (1)$$

where ω_{ij} is the phylogenetic/taxonomic path length between species *i* and *j*, and *S* is the number of species).

While the variation in taxonomic distinctness (Λ+) was calculated as follows:

$$\Lambda^+ = \left[\Sigma\Sigma_{i<j}\left(\omega_{ij} - \Delta^+\right)\right]/[S(S-1)/2] \qquad (2)$$

The average taxonomic distinctness (Δ^+) measures the average taxonomic distance between different species in an assemblage; the higher the values of Δ^+, the more significant the average taxonomic difference between species in the assemblage. Conversely, Λ^+ is the variation in branch lengths amongst all pairs of species and measures the distribution of branch lengths within a sample; the greater the variation in branch lengths, the greater the value of Λ^+.

To assess the differences in taxonomic distinctness (Δ^+) from expected Δ^+ values determined from the species list of polychaetes from the Gulf of Mexico, we used a randomization approach (as suggested by Clarke & Warwick (1998, 2001) and by Warwick & Clarke (2001)). A

simulated distribution was created, leading to a theoretical mean (a horizontal line displaying the taxonomic distinctness for all polychaete species shown in the graph of Δ^+ or Λ^+ for all the sampling stations against richness in each station) and to a confidence funnel for each, Δ^+, and Λ^+, from random subsamples of the polychaete species as suggested by Bhat and Magurran (2006). The simulation creates numerous random subsets of species, each of size *m*, from the entire species list, computes the corresponding Δ^+ and Λ^+ values, and establishes a range in which 95% of these values are included. Values of Δ^+ and Λ^+ located within the 95% probability funnel indicate that species diversity in the corresponding areas falls within the expected range, thus allowing both for sample size and effort-free diversity comparisons.

To evaluate the effects of geographical distance on taxonomic composition (Distance Decay), we calculated the distance in km between all pairs of sampling stations. Next, we used the geographical coordinates to calculate the distance between all pairs of sampling stations. Next, the relationship between pairwise faunal dissimilarity and spatial distance was assessed by fitting an exponential function describing the increase in faunal dissimilarity with spatial distance (Nekola & McGill, 2014; Nekola & White, 1999); the R package "betapart" (functions "decay.model" and "boot.coefs") was used; as it adjusts a General Linear Model (GLM) with dissimilarity as the response variable, spatial distance as the predictor, log link, and Gaussian error. Finally, the intercept and slope parameters were bootstrapped (1000 replicates) (Baselga et al., 2013; Gómez-Rodríguez & Baselga, 2018).

The indices were computed using the polychaete species' taxonomic hierarchy based on the Linnaean classification using the Plymouth Routines in Multivariate Ecological Research PRIMER-E (Clarke et al., 2014; Clarke & Gorley, 2015). The resulting matrices were examined to derive dissimilarity patterns through Ward's hierarchical cluster analysis on the taxonomic dissimilarity matrix. The optimal number of clusters was determined using the average silhouette width and the diagnostic group criteria suggested by Borcard et al. (2018). The cluster analysis was run on the θ^+ taxonomic dissimilarity index, a presence/absence "beta diversity" coefficient (Clarke et al., 2014; Clarke et al., 2006). The significant differences in the assemblage structure between the two studied regions were tested with a one-way analysis of similarity (ANOSIM).

In addition, we calculated the geographic distance between each pair of stations using the longitude and latitude coordinates of each station and the environmental distance between each

pair of stations with Euclidean distances based on all environmental variables measured. We used each sampling station's sediment composition, depth, temperature, and salinity as environmental variables. Distance-based Moran's eigenvector maps (dbMEMs), redundancy analysis (dbRDA), and variation partitioning were used to quantify the effect of spatial structure and biotic and abiotic variables on community dissimilarity (taxonomic distinctness). Before the dbRDA analysis, forward selection procedures were conducted to select the most significant spatial and environmental variables; only the dbMEMs corresponding to positive autocorrelation as spatial variables were used in the analyses. Distance-based Moran eigenvector maps (dbMEMs) for the matrix of the sites' geographical coordinates were used to describe the dataset's spatial structure. The initial dbMEMs model the large-scale spatial correlation, while the last dbMEMs correspond to fine-scale spatial correlations, which may capture variation at the sampling site scale. All non-significant ($P > 0.05$) variables were eliminated from further analyses. Afterward, we performed a variation partitioning analysis to assess the relative contribution of the significant spatial and environmental variables in explaining the community dissimilarity (Peres-Neto et al., 2006). The variation was partitioned into four fractions: (a) influence of environmental variables alone (sediment composition, depth, temperature, and salinity); (b) influence of spatial variables; (c) influence of spatial variables alone (distance-based Moran eigenvector maps; dbMEMs); and (d) unexplained variation. The variation partitioning analysis included only the variables selected by this procedure. The significance of each fraction was tested by Monte Carlo permutation tests. In addition, the relative contribution of each significant spatial and environmental variable in determining community dissimilarity was assessed by partial dbRDA to partial out the effect of each significant environmental/spatial variable. A Monte Carlo permutation test was run to test the significance of each significant spatial and environmental variable. All analyses were run in R-4.0.3 with the "vegan" and other built-in packages (Oksanen et al., 2020; R Development Core Team, 2020)

Results

3.1. Taxonomic Distinctness and Distance Decay

One hundred and seventy-three species from 40 families were recorded from the continental shelf of the Southern Gulf of Mexico. The eastern continental shelf region (Campeche Bank) had

the highest number of species (164). In contrast, 125 species were collected in the western region of the study area (Bay of Campeche).

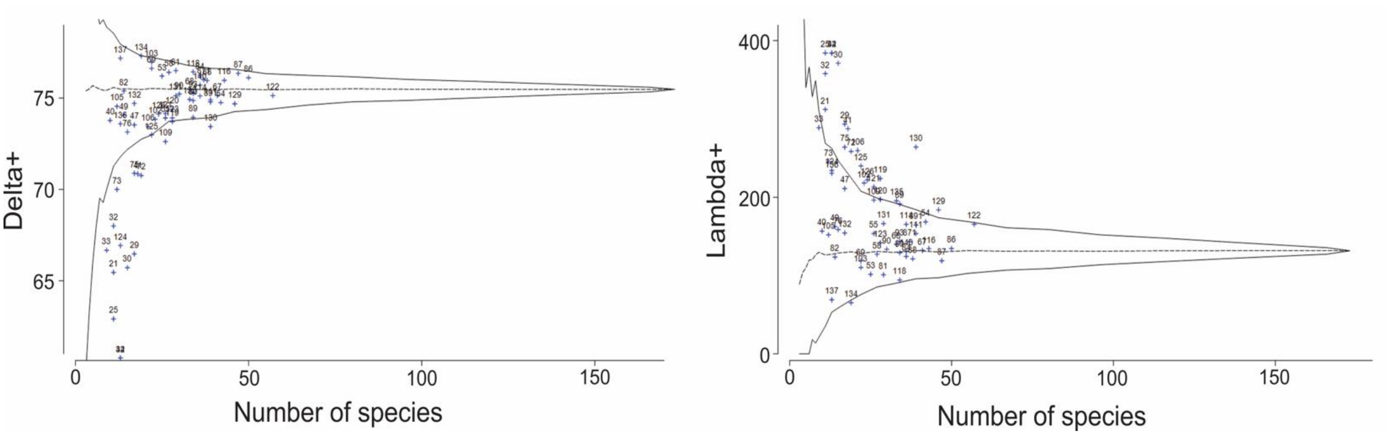

Figure 2. Simulated distribution of average taxonomic distinctness (Δ^+) and the variation in taxonomic distinctness Λ^+ (theoretical mean, horizontal, dashed line) for random subsets of species from the complete species list of 173 polychaete species from the Southern Gulf of Mexico and the 95% confidence limits (the funnel).

The estimated Δ^+ values resulting from the polychaete species list in each of the 61 sampling stations from the Southern Gulf of Mexico showed that most of the calculated Δ^+ values were close to the expected simulated mode of the funnel (Fig. 2). According to these Δ^+ values, most of the stations were as diverse as expected since they were located inside the funnel. However, a few sampling stations (21, 25, 29, 30, 32, 33, 34, 41, 42, 72, 73, 75, and 124), mainly located on the terrigenous shelf in the southernmost Gulf of Mexico, displayed lower diversity than expected.

The variation in taxonomic distinctness (Λ^+) showed that most stations were placed inside the funnel, close to the expected values. However, a few stations fell above the simulated funnel's upper limit, indicating higher-than-expected Λ^+ values (Fig. 2).

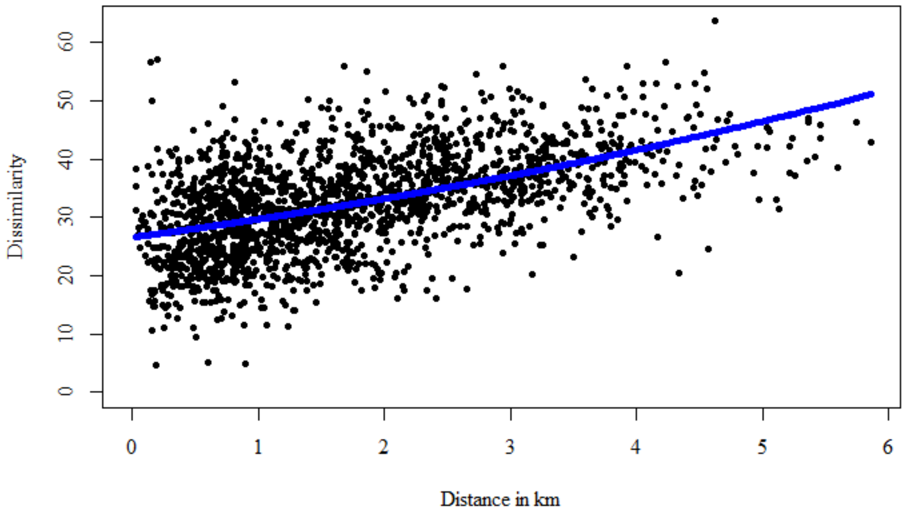

Figure 3. Taxonomic dissimilarity index θ^+ plotted against distance for all pairwise comparisons between sampling stations. The blue line represents the exponential function describing the increase in dissimilarity (a.intercept = 26.56; b.slope = 0.001).

The exponential fit between the calculated value of θ^+ taxonomic dissimilarity index as a function of the distance between all pairs of sampling stations showed an exponential relationship between compositional dissimilarity and distance. The distance among stations exhibited high variability, but most differed at less than 200 km. The dissimilarity between the polychaete taxonomic composition from all stations exponentially increased with increasing geographical distance (Figure 3).

3.2. Cluster Analysis

Ward's Clustering, using the θ^+ taxonomic dissimilarity index relating to the polychaete composition of the 61 sampling stations, showed three main faunal assemblages with high taxonomic dissimilarity (>0.75) between them (Figure 4). The ANOSIM analysis confirmed that spatial differences between the three polychaete assemblages were significant ($R_{ANOSIM(C1-C2)}$= 0.374, p= 0.001; $R_{ANOSIM(C1-C3)}$= 0.45, p= 0.001; $R_{ANOSIM(C2-C3)}$= 0.508, p= 0.001). The "Terrigenous assemblage" (Cluster 1 in red) was integrated by stations located in the southernmost

region of the terrigenous shelf of the Gulf of Mexico. Interestingly, sites 114, 126, and 140, associated with Cluster 1, are geographically located on the Carbonate Shelf. The "Transitional" assemblage (Cluster 2 in green) grouped the stations from the central part of the study area in front of Laguna de Términos, considered a sedimentary transitional region. Finally, the "Carbonated" assemblage (Cluster 3 in blue) included stations located on the carbonate shelf on the eastern Gulf of Mexico.

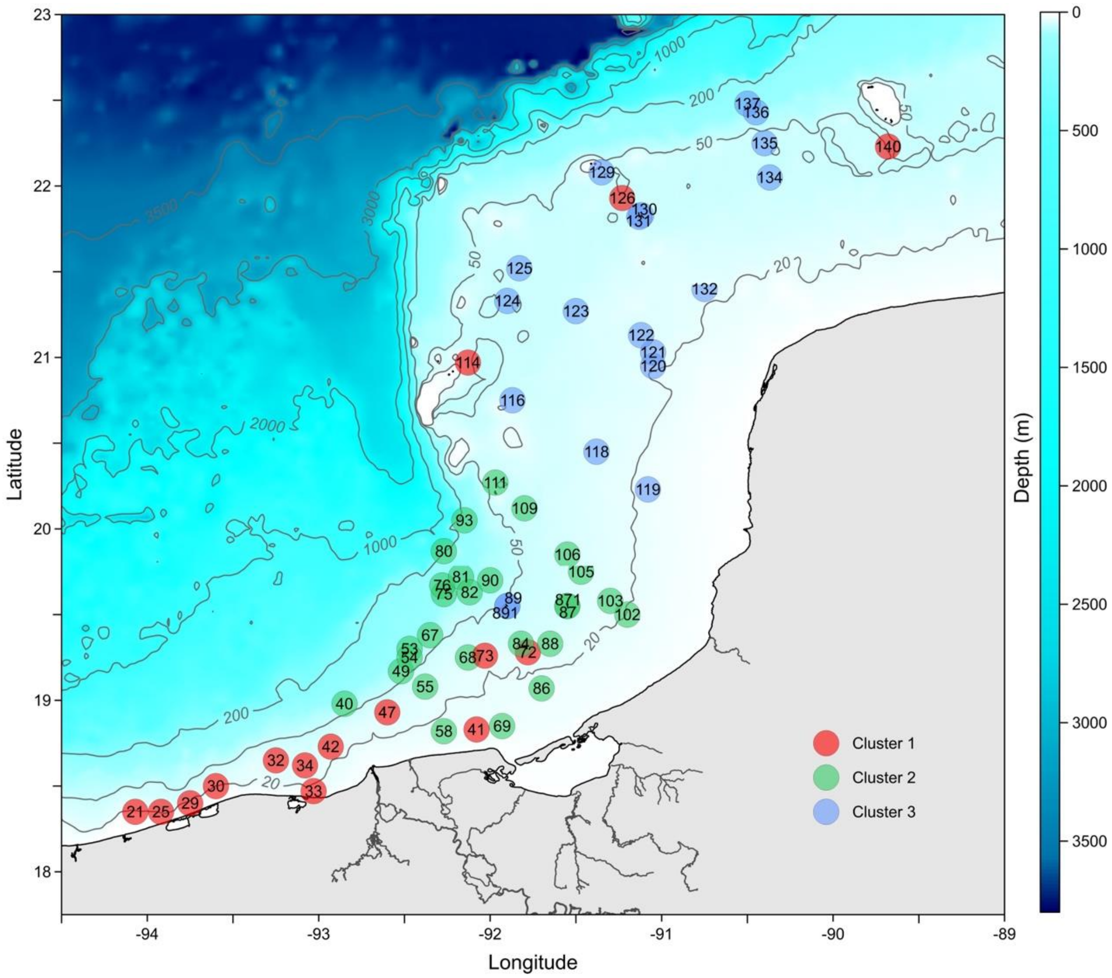

Figure 4. Cluster analysis based on taxonomic dissimilarity and Ward's clustering method

3.3. Distance-Based Redundancy Analysis (dbRDA)

The distance-based redundancy analysis (dbRDA) showed that the first axis explained 43.56% of the total variation, and the second explained 24.08% (Figure 5). The first axis represented a large-scale (with a high correlation with dbMEM1) sedimentary and thermal gradient

characterized by increasing sand content and temperature. The “Carbonate” assemblage (blue) in the eastern region was associated with warm water and sandy sediments. Stations belonging to the “Terrigenous” assemblage (red) in the western study area are associated with a medium-scale (dbMEM7) salinity gradient. The “Transitional” assemblage (green) in front of Laguna de Términos was related to salinity variability and increasing mud content in the sediment (Figure 5). All the environmental variables seem to be spatially structured. The unexplained variation may be linked to describers not used in the present study or simply due to random factors.

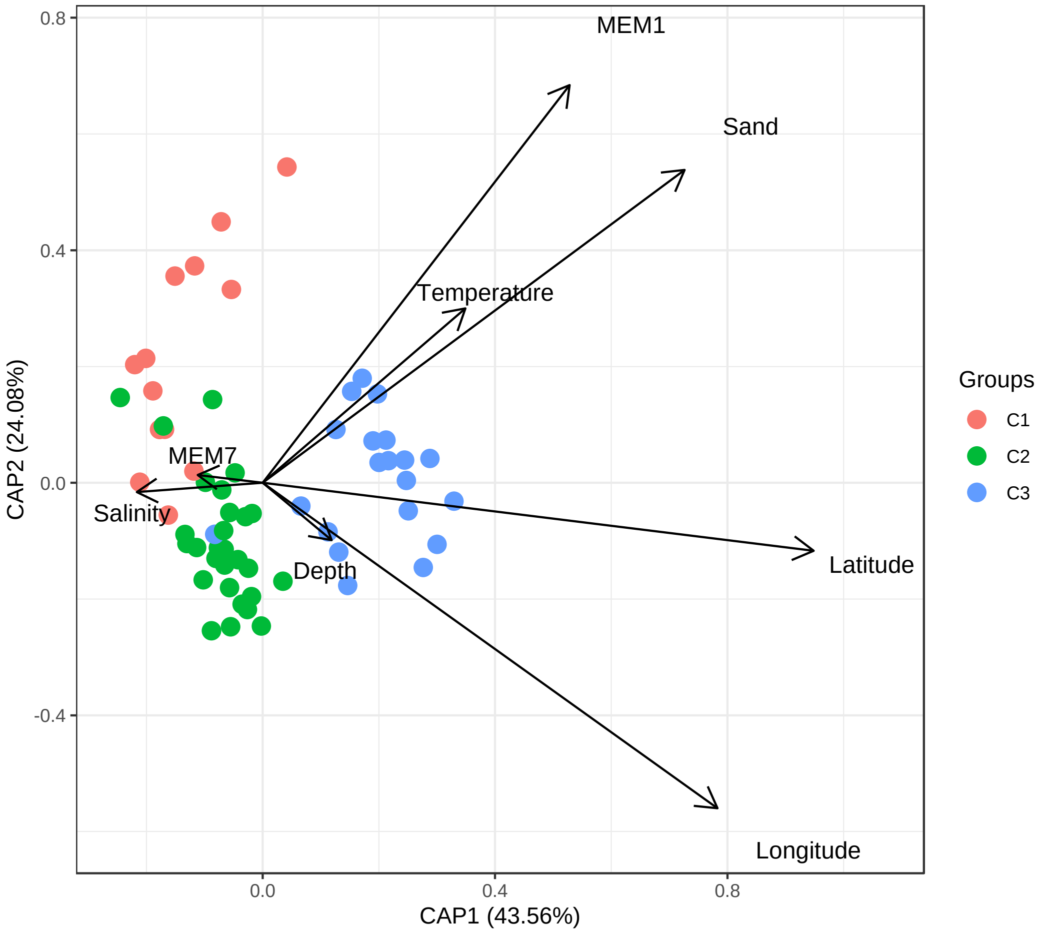

Figure 5. Distance-based redundancy analysis (dbRDA) ordination plot showing the relationships between sampling stations based on polychaete taxonomic distinctness and spatial and environmental constraints.

3.4. Variation Partitioning

The set of environmental (X1) and spatial variables (dbMEM1, X3) explains 5% of the variation in taxonomic dissimilarity across the study area. The environmental variables alone (X1 in the partitioning results) explain almost 10% of the variation, of which 5% is not spatially structured. This fraction represents species-environment relationships associated with local environmental conditions. Medium-scale spatial processes (dbMEM7, X4) explained 3.4% of the taxonomic variability. Finally, the linear spatial structure (x and y coordinates; X2) contributions were less significant (1.5%) (Figure 6).

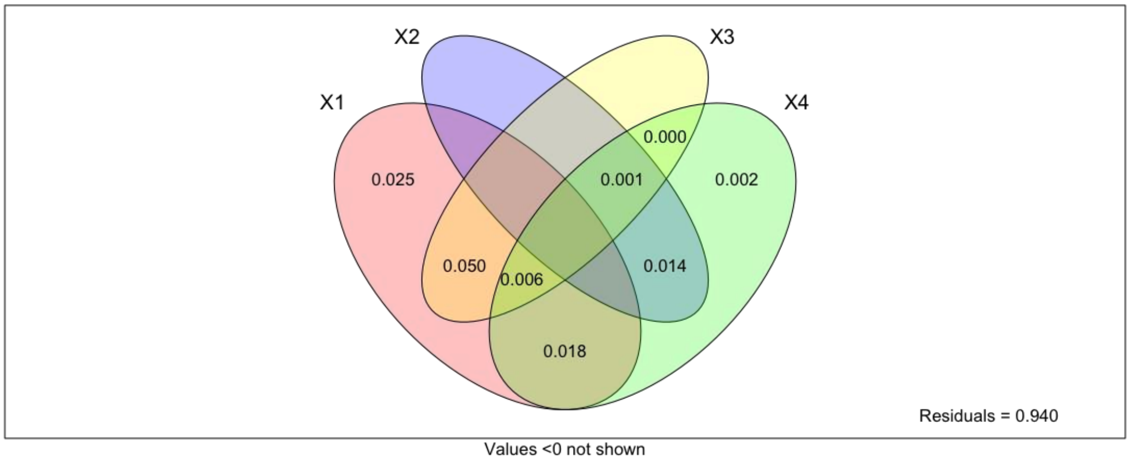

Figure. 6. Variation partitioning of the taxonomic dissimilarity into an environmental component (X1), linear spatial processes (X2), a broad-scale (X3), and fine-scale (X4) dbMEM spatial components.

Discussion

In the Southern Gulf of Mexico, intensive oil extraction and other hydrocarbons have been carried out since the 70s; thus, the ability to assess this industry's naturally polluting and associated anthropic impacts is important (Hernández-Arana et al., 2003, 2005). Therefore, understanding the distributional patterns of the polychaetes species present can help us understand the ecological processes occurring in the benthic ecosystems and how these organisms could be used as indicators

of different ecological conditions at a larger scale (Gambi & Giangrande, 1986; Quiroz-Martínez et al., 2021).

In the Southern Gulf of Mexico, polychaete taxonomic distinctness changed across the longitudinal gradient, increasing from west to east, linked to a decline in species richness towards the terrigenous shelf in the west. The low taxonomic distinctness observed in terrigenous environments could be related to its more homogenous conditions, allowing the coexistence of taxonomically close species. In contrast, in the carbonated environment, the habitats are more varied, with highly heterogenous bottoms having different grain sizes, enabling the occurrence of taxonomically distinct species with different ecological preferences. Sediment composition is one of the main factors influencing the settling and distribution of benthic species. Sediment type and grain size variations determine organic matter content, dissolved gases, and nutrient concentrations (Granados Barba, 2001; Gray, 1974, 1981; Magni et al., 2004, 2008, 2009). The mixed sandy-mud bottoms dominate the Campeche Bank, favoring higher densities and species richness. This contrasts with the terrigenous sediments from the western Gulf, which negatively affect the benthic communities' diversity. Our analyses allowed us to distinguish the occurrence of three distinct polychaete species assemblages with the sedimentary distribution in the southern Gulf: one located in the western region on the terrigenous shelf, another associated with the carbonate shelf to the east, and an intermediate zone, in front of Laguna de Términos.

In the Bay of Campeche, the circulation is dominated by the mesoscale cyclonic eddy, which develops in the eastern bay and moves westward, enhancing small-scale anticyclonic and cyclonic eddies (Salas-de-León et al., 2008). In the study area, a coastal current moves, southward or south-westward, along the east coast mainly; approximately at 93°50W longitude, it converges with an eastward current along the coast to form a northward current along the shelf break (Salas-de-León et al., 2008). The sediment distribution in the southern Gulf of Mexico (Bay of Campeche) is mainly driven by wind-induced currents and mesoscale cyclonic eddies (Salas-de-León et al., 2008). Coarse sediments are located near the coast in the convergence region and the core of the anticyclonic eddy. In turn, fine sediments are situated off the shelf coast and in areas where the weakest currents are recorded (Salas-de-León et al., 2008). Carbonates dominate sediments on the eastern side of the Bay of Campeche since no river discharges exist or any other systems that could

contribute terrigenous sediments, whereas, in the southernmost part of the bay, where major rivers are located, the bottom sediments are mainly of terrigenous origin (Salas-de-León et al., 2008).

The composition and structure of benthic assemblages can be influenced by numerous physical and biological factors (Hernández-Alcántara et al., 2011; Warwick, 1984) that define their zonation along environmental gradients (Bergen et al., 2001). In the study area, sediment variations were essential in structuring the polychaete assemblages. In addition, bathymetric fluctuations usually promote changes in other depth-dependent physical variables (Carvalho et al., 2012; Gray, 1981; Probert et al., 2001; Van Hoey et al., 2004). Particularly in the Southern Gulf, the distance between the sampling stations and their geographical proximity directly affected the faunal differences since the dissimilarity in polychaete species composition was a function of the distance separating them. Distance decay curves at regional or local scales are primarily shaped by the organisms' biological characteristics (niche requirements, dispersal ability) and environmental interactions. Nearby locations should typically have highly different species compositions when a species has a narrow geographic range, and power-law or negative exponential functions can be used to approximate distance-decay curves. However, close-by biological communities may be very similar when species have large spatial ranges relative to the study extent. This leads to a low or zero rate of change in community composition at the closest distances, while the actual decrease in community similarity would start at intermediate distances (Martín-Devasa et al., 2022). Several studies have shown that sediment composition can be crucial in controlling benthic communities in marine environments (Snelgrove & Butman, 1994). Notably, in the Gulf of Mexico, it has been demonstrated that sediment type and depth were leading factors in determining polychaete species' distribution and abundance patterns. Granados-Barba (2001) and Quiroz-Martínez et al. (2021, 2022) also showed that, in the southern Gulf, the polychaete species richness increased with depth. Several studies have shown that depth is the major factor in structuring macrofauna (Hyland et al., 1991; Oug et al., 2017).

In contrast, in other studies, it has been found that sediment composition is the main factor affecting the benthic fauna (Hernández-Alcántara et al., 2011; Quiroz-Martínez et al., 2021; Snelgrove & Butman, 1994). In this sense, our results, founded on distance-based Redundancy analysis, showed that sediment type (and the dbMEMs) were the primary factor, rather than depth, to determine the polychaete faunal structure in the Southern Gulf of Mexico. For example, the

syllids, usually small and highly motile, constituted the most diverse polychaete family and were mainly found in carbonated bottoms, where coarse sands mixed with coral and shell rubble dominate. However, the syllids become uncommon in mud and sandy-mud terrigenous sediments. In the soft bottoms of the eastern Campeche Bay, syllids species richness generally followed a decreasing gradient from carbonate to terrigenous sediments (east to west) (Granados Barba et al., 2003). In addition, dbMEMs spatial processes explained a higher proportion of the variance in polychaete community structure than the environmental variables. Although polychaete larvae can disperse long distances, adult dispersal is often restricted and occurs over local/small spatial scales, mainly in intertidal areas and during local disturbance events (Checon & Amaral, 2017). Our results suggest that species composition was spatially correlated at shorter distances and that dispersal limitation and species response to spatially structured environmental gradients might be involved in determining polychaete distribution patterns.

The observed changes in sediment type affecting the establishment and development of benthic organisms can be associated with the water-sediment interphase dynamics that modify the organisms' excavation processes and limit the number of species that can settle (Díaz-Castañeda & Harris, 2004). According to Santibañez-Aguascalientes et al. (2020), the carbonate bottoms to the east comprise medium and fine calcarenites and oxides that provide suitable habitats for benthic communities (Carranza-Edwards et al., 1993; Cuevas Madrid et al., 2018; Granados Barba et al., 2003). In turn, the terrigenous bottoms are influenced by fluvial discharges dragging mud and sand sediments and harbor a high diversity of polychaete species adapted to turbid bottoms and terrigenous material (Hernández-Alcántara et al., 2011; Santibañez-Aguascalientes et al., 2018).

Characterizing distinct groups of invertebrates can be a complex process since, along biological gradients, a gradual transition between different assemblages frequently occurs (Gray, 1981; Van Hoey et al., 2004). In the present study, the separation of species groups based on multivariate analysis and taxonomic distinctness allowed us to distinguish three large polychaete assemblages; this indicates a gradual change in the composition of the polychaete fauna along the longitudinal gradient. Furthermore, polychaete assemblages distributed in the transitional region were different from the other sedimentary environments; this could be related to the high environmental variability of the transitional area, characterized by the transition from terrigenous

to carbonated sediments, modulated by the Grijalva-Usumacinta plume and the oil extraction activities that affect the benthic fauna by reducing species diversity and richness (Salas-de-León et al., 2008; Santibañez-Aguascalientes et al., 2021, 2023). Ecological research in the Southern Gulf of Mexico shows that "Nortes" and summer rains provoking high river run-off are the most important physical processes influencing large-scale benthic community structure across the carbonate and terrigenous shelves (Cornell & Karlson, 2000; Granados Barba et al., 2003; Hernández-Arana et al., 2003, 2005; Soto & Escobar-Briones, 1995; Yáñez-Arancibia & Sánchez-Gil, 1983).

Finally, connectivity is another process that could explain the results presented in this study. Connectivity is an essential process in any marine ecosystem because it influences, among other things, the colonization of habitats, the maintenance of environmental services, and the persistence and survival of marine benthic populations (Gerber et al., 2014). According to Treml et al. (2015), there are 4-stage processes that explain the connectivity between populations; the second one (their Figure 1) includes the dispersion, transport, and movement of the organisms, which is strongly determined by the advection potential, which in turn is related to the magnitude of the currents. This could explain the three distinct faunal assemblages observed in this study, considering that the Southern Gulf of Mexico is highly dynamic, with the confluence of mesoscale physical processes and intense coastal currents, as mentioned above, which could have influenced the polychaete patterns of distribution presented in this study. For example, the connectivity between coral reef systems from the Gulf of Mexico has already been demonstrated using biological data (Carricart-Ganivet, 2004; Johnston & Akins, 2016; Mumby & Hastings, 2007; Murphy et al., 1999; Ortiz-Lozano et al., 2013). In addition, Salas-Monreal et al. (2018) elucidated the different pathways that connect the Western Gulf of Mexico reef systems using monthly geostrophic velocities, sea surface temperature, and chlorophyll-*a* values. They found high connectivity between the reef systems that bring substrates, suspended matter, and organisms from the Campeche Reef System (CRS) in the Campeche Bank to the Veracruz Reef System (VRS) in the Southwestern Gulf of Mexico. According to them, the geostrophic current velocities suggested various paths connecting the various coral reef habitats, with two open ocean pathways in addition to the traditional coastal alongshore path, which is constrained to the continental shelf, with the first linking the Campeche Reef System with the Veracruz System. The water circulation pattern is driven by the Caribbean current during the spring and summer, with south to southwestern

direction; however, during the autumn-winter seasons, the flow reverts to the east to a north-eastern direction (Boicourt et al., 1998; Salas-Monreal et al., 2018). Various studies on connectivity in the southern Gulf of Mexico indicate a low east-west flow from the Campeche Bank to the Veracruz reefs; connectivity is stronger going west to east (Sanvicente-Añorve et al., 2014). However, ocean currents in this region are complex, with eddies and seasonal shifts in direction within the inner shelf, showing fluxes from east to west during summer and west to east during winter (Salas-Monreal et al., 2018). The pattern of taxonomic distribution observed in this study suggests connectivity between the reef systems

In this study, the environment was characterized by spatial processes (dbMEMs), temperature, depth, salinity, and sediment variability; however, other variables, such as concentration of organic matter or nutrients, influence the distribution of polychaetes. Therefore, further studies should explore whether these relationships may produce higher percentages of variance explained by environmental structuring.

Acknowledgments

Thanks are due to all the participants (students, researchers, and crew) of the research expeditions “IMCA” and “DINAMO”, carried out on board the R/V “Justo Sierra” (Universidad Nacional Autónoma de México) during which the biological material used in this study was collected. We also thank all the students of the Laboratorio de Ecología y Biodiversidad de Invertebrados Marinos of the Instituto de Ciencias del Mar y Limnología (ICML), UNAM, for their valuable assistance in the processing of samples. This research was funded by the Universidad Nacional Autónoma de México DGAPA/PAPIIT Project IA202321 “Análisis y visualización de bases de datos de biodiversidad en sistemas marinos para determinar cambios en los patrones de distribución de la riqueza de especies y la abundancia de la fauna marina del Golfo de México” and through grant 628 from the Instituto de Ciencias del Mar y Limnología, Universidad Nacional Autónoma de México. We are grateful to Jorge Castro for improving the figures.

Aldeco-Ramírez, J., Monreal-Gómez, M. A., Signoret, M., Salas-de-León, D. A., & Hernández-Becerril, D. U. (2009). Occurrence of a subsurface anticyclonic eddy, fronts, and *Trichodesmium* spp. *Ciencias Marinas*, *35*(4), 333–344. https://doi.org/https://doi.org/10.7773/cm.v35i4.1551

Alves, A. T., Petsch, D. K., & Barros, F. (2020). Drivers of benthic metacommunity structure along tropical estuaries. *Scientific Reports*, *10*(1), 1739. https://doi.org/10.1038/s41598-020-58631-1

Antoine, J. (1972). Structure of the Gulf of Mexico. In R. Rezak & V. J. Henry (Eds.), *Contributions on the geological and geophysical oceanography of the Gulf of Mexico* (Vol. 3, pp. 1–34). Texas A&M University.

Arvanitidis, C., Bellan, G., Drakopoulos, P., Valavanis, V., Dounas, C., Koukouras, A., & Eleftheriou, A. (2002). Seascape biodiversity patterns along the Mediterranean and the Black Sea: lessons from the biogeography of benthic polychaetes. *Marine Ecology Progress Series*, *244*, 139–152.

Baselga, A., Orme, C. D. L., & Villéger, S. (2013). *betapart: Partitioning beta diversity into turnover and nestedness components* (R package version 1.5.4). Art. R package version 1.5.4. https://cran.r-project.org/package=betapart

Belan, T. A. (2003). Marine envirnonmental quality assessement using polychaete taxocene characteristics in Vancouver harbour. *Marine Environmental Research*, *57*, 89–101.

Benedetti-Cecchi, L., Iken, K., Konar, B., Cruz-Motta, J., Knowlton, A., Pohle, G., Castelli, A., Tamburello, L., Mead, A., Trott, T., Miloslavich, P., Wong, M., Shirayama, Y., Lardicci, C., Palomo, G., & Maggi, E. (2010). Spatial Relationships between Polychaete Assemblages and Environmental Variables over Broad Geographical Scales. *Plos One*, *5*(9), 1–10.

Bergen, M., Weisberg, S., Smith, R., Cadien, D., Dalkey, A., Montagne, D., Stull, J., Velarde, R., & Ranasinghe, J. (2001). Relationship between depth, sediment, latitude and the structure of benthic infaunal assemblages on the mainland shelf of southern California. *Marine Biology*, *138*, 637–647.

Bhat, A., & Magurran, A. E. (2006). Taxonomic distinctness in a linear system: a test using a tropical freshwater fish assemblage. *Ecography*, *29*, 104–110. https://doi.org/10.1111/j.2006.0906-7590.04418.x

Boicourt, W. C., Wiseman Jr., W. J., Valle-Levinson, A., & Atkinson, L. P. (1998). Continental shelf of the southeastern United States and the Gulf of Mexico: In the shadow of the western boundary current. In A. R. Robinson & K. H. Brink (Eds.), *The sea. The Global Coastal Ocean. Regional studies and syntheses* (pp. 135–182). John Wiley.

Borcard, D., Gillet, F., & Legendre, P. (2018). *Numerical Ecology with R* (2nd Edition). Springer International Publishing. https://doi.org/10.1007/978-3-319-71404-2

Borcard, D., Legendre, P., & Drapeau, P. (1992). Partialling out the Spatial Component of Ecological Variation. *Ecology*, *73*(3), 1045–1055. https://doi.org/10.2307/1940179

Capa, M., & Hutchings, P. (2021). Annelid Diversity: Historical Overview and Future Perspectives. *Diversity*, *13*(3), 1–14. https://doi.org/https://doi.org/10.3390/d13030129

Carranza-Edwards, A., Rosales-Hoz, L., & Monreal-Gómez, M. A. (1993). Suspended sediments in the southeastern Gulf of Mexico. *Marine Geology*, *112*, 257–259.

Carricart-Ganivet, J. P. (2004). Sea surface temperature and the growth of the West Atlantic reef-building coral *Montastraea annularis*. *Journal of Experimental Marine Biology and Ecology*, *302*(2), 249–260. https://doi.org/10.1016/j.jembe.2003.10.015

Carvalho, S., Cunha, M. R., Pereira, F., Pousão-Ferreira, P., Santos, M. N., & Gaspar, M. B. (2012). The effect of depth and sediment type on the spatial distribution of shallow soft-bottom amphipods along the southern Portuguese coast. *Helgoland Marine Research*, *66*(4), 489–501. https://doi.org/10.1007/s10152-011-0285-9

Chase, J. M., & Leibold, M. A. (2003). *Ecological Niches*. University of Chicago Press. https://doi.org/10.7208/chicago/9780226101811.001.0001

Checon, H. H., & Amaral, A. C. Z. (2017). Taxonomic sufficiency and the influence of rare species on variation partitioning analysis of a polychaete community. *Marine Ecology*, *38*(1), e12384. https://doi.org/https://doi.org/10.1111/maec.12384

Clarke, K., Gorley, R. N., Somerfield, P. J., & Warwick, R. M. (2014). *Change in marine communities: an approach to statistical analysis and interpretation*. PRIMER-E.

Clarke, K. R., & Gorley, R. N. (2015). *Primer v7: user manual/tutorial*. 300.

Clarke, K. R., Somerfield, P. J., & Chapman, M. G. (2006). On resemblance measures for ecological studies, including taxonomic dissimilarities and a zero-adjusted Bray–Curtis coefficient for denuded assemblages. *Journal of Experimental Marine Biology and Ecology*, *330*, 55–80.

Clarke, K. R., & Warwick, R. M. (1998). A taxonomic distinctness index and its statistical properties. *Journal of Applied Ecology*, *35*, 523–531.

Clarke, K. R., & Warwick, R. M. (2001). A further biodiversity index applicable to species lists: variation in taxonomic distinctness. *Marine Ecology Progress Series*, *216*, 265–278.

Cornell, H. V, & Karlson, R. H. (2000). Coral species richness: ecological versus biogeographical influences. *Coral Reefs*, *19*(1), 37–49. https://doi.org/https://doi.org/10.1007/s003380050224

Cuevas Madrid, H., Rosales Hoz, L., Márquez García, A. Z., & Carranza Edwards, A. (2018). Environmental assessment of a mangrove protected area in the southeast Gulf

of Mexico through sediment core analysis. *Environmental Earth Sciences*, *77*(3), 73. https://doi.org/10.1007/s12665-017-7160-1

Dauvin, J. C., & Bellan-Santini, D. (2004). Biodiversity and the biogeographic relationships of the Amphipoda: Gammaridea on the French coastline. *Journal of the Marine Biological Association of the United Kingdom*, *84*, 621–628.

Díaz-Castañeda, V., & Harris, L. H. (2004). Biodiversity and structure of the polychaete fauna from soft bottoms of Bahia Todos Santos, Baja California, Mexico. *Deep-Sea Research II*, *51*, 827–847.

Dogan, A., Çinar, M. E., Onen, M., Ergen, Z., & Katagan, T. (2005). Seasonal dynamic of soft-bottom zoobenthic communities in polluted and unpolluted areas of Izmir Bay (Aegean Sea). *Senckenbergiana Maritima*, *35*, 133–145.

Dorr, B. S., Hanson-Dorr, K. C., Assadi-Porter, F. M., Selen, E. S., Healy, K. A., & Horak, K. E. (2019). Effects of Repeated Sublethal External Exposure to Deep Water Horizon Oil on the Avian Metabolome. *Scientific Reports*, *9*(1), 371. https://doi.org/https://doi.org/10.1038/s41598-018-36688-3

Dray, S., Legendre, P., & Peres-Neto, P. R. (2006). Spatial modelling: a comprehensive framework for principal coordinate analysis of neighbour matrices (PCNM). *Ecological Modelling*, *196*(3), 483–493. https://doi.org/https://doi.org/10.1016/j.ecolmodel.2006.02.015

Dray, S., Pélissier, R., Couteron, P., Fortin, M.-J., Legendre, P., Peres-Neto, P. R., Bellier, E., Bivand, R., Blanchet, F. G., De Cáceres, M., Dufour, A.-B., Heegaard, E., Jombart, T., Munoz, F., Oksanen, J., Thioulouse, J., & Wagner, H. H. (2012). Community ecology in the age of multivariate multiscale spatial analysis. *Ecological Monographs*, *82*(3), 257–275. https://doi.org/https://doi.org/10.1890/11-1183.1

Economo, E. P., & Keitt, T. H. (2008). Species diversity in neutral communities: a network approach. *Ecology Letters*, *11*, 52–62.

Ergen, Z., Çinar, M. E., Dagli, E., & Kurt, G. (2006). Seasonal dynamics of Soft-Bottom Polychaetes in Izmir Bay (Aegean Sea, Eastern Mediterranean). *Scientia Marina*, *70S3*, 197–207.

F. Dormann, C., M. McPherson, J., B. Araújo, M., Bivand, R., Bolliger, J., Carl, G., G. Davies, R., Hirzel, A., Jetz, W., Daniel Kissling, W., Kühn, I., Ohlemüller, R., R. Peres-Neto, P., Reineking, B., Schröder, B., M. Schurr, F., & Wilson, R. (2007). Methods to account for spatial autocorrelation in the analysis of species distributional data: a review. *Ecography*, *30*(5), 609–628. https://doi.org/https://doi.org/10.1111/j.2007.0906-7590.05171.x

Fauchald, K., Granados-Barba, A., & Solís-Weiss, V. (2009). Polychaeta (Annelida) of the Gulf of Mexico. *Gulf of Mexico Origin, Waters, and Biota*, *1*, 751–788.

Fuentes-Yaco, C., León, D. A. S. de, Monreal-Gómez, M. A., & Vera-Herrera, F. (2001). Environmental forcing in a tropical estuarine ecosystem: the Palizada River in the southern Gulf of Mexico. *Marine and Freshwater Research*, *52*(5), 735–744. https://doi.org/https://doi.org/10.1071/MF00077

Gambi, M. C., & Giangrande, A. (1986). Distribution of soft-bottom polychaetes in two coastal areas of the Tyrrhenian Sea (Italy): Structural Analysis. *Estuarine, Coastal and Shelf Science*, *23*(6), 847–862.

Gerber, L. R., Mancha-Cisneros, M. D. M., O'Connor, M. I., & Selig, E. R. (2014). Climate change impacts on connectivity in the ocean: Implications for conservation. *Ecosphere*, *5*(3), art33. https://doi.org/10.1890/ES13-00336.1

Giangrande, A., & Gravina, M. F. (2015). Brackish-water polychaetes, good descriptors of environmental changes in space and time. *Transitional Waters Bulletin*, *9*, 42–55.

Giangrande, A., Licciano, M., & Musco, L. (2005). Polychaetes as environmental indicators revisited. *Marine Pollution Bulletin*, *50*(11), 1153–1162. https://doi.org/https://doi.org/10.1016/j.marpolbul.2005.08.003

Gilbert, B., & Bennett, J. R. (2010). Partitioning variation in ecological communities: do the numbers add up? *Journal of Applied Ecology*, *47*(5), 1071–1082. https://doi.org/https://doi.org/10.1111/j.1365-2664.2010.01861.x

Glasby, C. J., & Read, G. (1998). Polychaete worms: a diverse yet poorly known group. *Water and Atmosphere*, *6*(4), 7–9.

Gómez-Rodríguez, C., & Baselga, A. (2018). Variation among European beetle taxa in patterns of distance decay of similarity suggests a major role of dispersal processes. *Ecography*, *41*(11), 1825–1834. https://doi.org/https://doi.org/10.1111/ecog.03693

Gopal, A., Abdul Jaleel, K. U., Parameswaran, U. V, Sanjeevan, V. N., Saramma, A. V, Vijayan, A., Saravanane, N., Gupta, G. V. M., & Sudhakar, M. (2020). Distinctive Community Patterns With Exceptional Diversity of Polychaetes Around a Tectonically Active Archipelago in the Tropical Indian Ocean. In *Frontiers in Marine Science* (Vol. 7). https://doi.org/https://doi.org/10.3389/fmars.2020.00710

Granados Barba, A. (2001). *Los poliquetos benticos de la región petrolera del suroeste del Golfo de México: Estructura comunitaria e impacto ambiental*.

Granados Barba, A., Solís-Weiss, V., Tovar-Hernández, M. A., & Ochoa-Rivera, V. (2003). Distribution and diversity of the Syllidae (Annelida: Polychaeta) from Mexican Gulf of Mexico and Caribbean. *Hydrobiologia*, *496*, 337–345.

Gray, J. S. (1974). Animal-sediment relationships. *Oceanography and Marine Biology: An Annual Review*, *12*, 223–262.

Gray, J. S. (1981). *The ecology of Marine Sediments. An introduction to the structure and function of benthic communities*. Cambridge University Press.

Heino, J., Grönroos, M., Soininen, J., Virtanen, R., & Muotka, T. (2012). Context dependency and metacommunity structuring in boreal headwater streams. *Oikos, 121*(4), 537–544. http://www.jstor.org/stable/41429322

Hernandez Arana, H. A., Attrill, M. J., Hartley, R., & Gold Bouchot, G. (2005). Transitional carbonate-terrigenous shelf sub-environments inferred from textural characteristics of surficial sediments in the Southern Gulf of Mexico. *Continental Shelf Research*, *25*(15), 1836–1852. https://doi.org/https://doi.org/10.1016/j.csr.2005.06.007

Hernández-Alcántara, P., Cortés-Solano, J. D., & Solís-Weiss, V. (2011). Polychaete diversity in lagoons and estuaries of the Southern Gulf of Mexico. In E. Özhan (Ed.), *Proceedings of the Tenth International Conference on the Mediterranean Coastal Environmen*.

Hernández-Arana, H. A., Rowden, A. A., Attrill, M. J., Warwick, R. M., & Gold-Bouchot, G. (2003). Large-scale environmental influences on the benthic macroinfauna of the southern Gulf of Mexico. *Estuarine, Coastal and Shelf Science*, *58*, 825–841.

Hernández-Arana, H. A., Warwick, R. M., Attrill, M. J., Rowden, A. A., & Gold-Bouchot, G. (2005). Assessing the impact of oil-related activities on benthic macroinfauna assemblages of the Campeche shelf, southern Gulf of Mexico. *Marine Ecology Progress Series*, *289*, 89–107. https://www.int-res.com/abstracts/meps/v289/p89-107/

Hutchings, P. A. (1998). Biodiversity and functioning of polychaete in benthic sediments. *Biodiversity and Conservation*, *7*, 1133–1145.

Hyland, J., Baptiste, H., Campbell, J., Kennedy, J., Kropp, R., & Williams, S. (1991). Macroinfaunal communities of the Santa Maria basin on the California outer continental shelf and slope. *Marine Ecology Progress Series*, *78*, 147–161.

Johnston, M. W., & Akins, J. L. (2016). The non-native royal damsel (Neopomacentrus cyanomos) in the southern Gulf of Mexico: An invasion risk? *Marine Biology*, *163*(1), 12. https://doi.org/10.1007/s00227-015-2777-7

Keitt, T. H., Bjørnstad, O. N., Dixon, P. M., & Citron-Pousty, S. (2002). Accounting for spatial pattern when modeling organism-environment interactions. *Ecography*, *25*(5), 616–625. https://doi.org/https://doi.org/10.1034/j.1600-0587.2002.250509.x

Knox, G. (1977). The role of polychaetes in benthic soft-bottom communities. In D. J. Reish & K. Fauchald (Eds.), *Essays on Polychaetous Annelids in memory of Dr Olga Hartman* (pp. 547–604). Allan Hancock Foundation, University of Southern California.

Legendre, P. (1993). Spatial Autocorrelation: Trouble or New Paradigm? *Ecology*, *74*(6), 1659–1673. https://doi.org/https://doi.org/10.2307/1939924

Legendre, P. (2018). Numerical ecology. In E. S. BV. (Ed.), *Encyclopedia of Ecology* (second Eng, Vol. 3). Elsevier Science BV. https://doi.org/10.1016/B978-0-12-409548-9.10595-0

Legendre, P., Borcard, D., & Peres-Neto, P. R. (2005). Analyzing Beta Diversity: Partitioning the Spatial Variation of Community Composition Data. *Ecological Monographs*, *75*(4), 435–450. https://doi.org/https://doi.org/10.1890/05-0549

Levin, L. A., Etter, R. J., Rex, M. A., Gooday, A. J., Smith, C. R., Pineda, J., Stuart, C. T., Hessler, R. R., & Pawson, D. (2001). Environmental Influences on Regional Deep-Sea Species Diversity. *Annual Review of Ecology and Systematics*, *32*(1), 51–93. https://doi.org/10.1146/annurev.ecolsys.32.081501.114002

López-Cabello, Z., Coria-Monter, E., Monreal-Gómez, M. A., Salas-de-León, D. A., Durán-Campos, E., & Gracia, A. (2022). The holoplanktonic mollusks (Pteropoda and Pterotrachoidea) in surface waters of the Campeche Canyon (southern Gulf of Mexico) during a winter storm (“Nortes”) season. *Check List*, *18*(6), 1321–1331. https://doi.org/10.15560/18.6.1321

Mackie, A. S. Y., Parmiter, C., & Tong, L. K. Y. (1997). Distribution and diversity of Polychaeta in the southern Irish Sea. *Bulletin of Marine Science*, *60*(2), 467–481.

Magni, P., De Falco, G., Como, S., Casu, D., Floris, A., Petrov, A. N., Castelli, A., & Perilli, A. (2008). Distribution and ecological relevance of fine sediments in organic-enriched lagoons: The case study of the Cabras lagoon (Sardinia, Italy). *Marine Pollution Bulletin*, *56*(3), 549–564. https://doi.org/https://doi.org/10.1016/j.marpolbul.2007.12.004

Magni, P., Micheletti, S., Casu, D., Floris, A., De Falco, G., & Castelli, A. (2004). Macrofaunal community structure and distribution in a muddy coastal lagoon. *Chemistry and Ecology*, *20*(sup1), 397–409. https://doi.org/https://doi.org/10.1080/02757540310001629161

Magni, P., Tagliapietra, D., Lardicci, C., Balthis, L., Castelli, A., Como, S., Frangipane, G., Giordani, G., Hyland, J., Maltagliati, F., Pessa, G., Rismondo, A., Tataranni, M., Tomassetti, P., & Viaroli, P. (2009). Animal-sediment relationships: Evaluating the ‘Pearson–Rosenberg paradigm’ in Mediterranean coastal lagoons. *Marine Pollution Bulletin*, *58*(4), 478–486. https://doi.org/https://doi.org/10.1016/j.marpolbul.2008.12.009

Martin, R. G., & Bouma, A. H. (1978). Physiography of the Gulf of Mexico. In A. H. Bouma, G. T. Moore, & J. M. Coleman (Eds.), *Framework, Fades, and Oil-Trapping Characteristics of the Upper Continental Margin* (Vol. 7, pp. 3–19). American Association of Petroleum Geologists.

Martín-Devasa, R., Martínez-Santalla, S., Gómez-Rodríguez, C., Crujeiras, R. M., & Baselga, A. (2022). Species range size shapes distance-decay in community similarity.

Diversity and Distributions, *28*(7), 1348–1357. https://doi.org/https://doi.org/10.1111/ddi.13550

Mikac, B., Licciano, M., Jaklin, A., Iveša, L., Giangrande, A., & Musco, L. (2020). Diversity and Distribution Patterns of Hard Bottom Polychaete Assemblages in the North Adriatic Sea (Mediterranean). In *Diversity* (Vol. 12, Issue 10). https://doi.org/10.3390/d12100408

Monreal-Gómez, M. A., Salas-de-León, D. A., Padilla-Pilotze, A. R., & Alatorre-Mendieta, M. A. (1992). Hydrography and estimation of density currents in the southern part of the Bay of Campeche, Mexico. *Ciencias Marinas*, *18*(4), 115–133.

Mumby, P. J., & Hastings, A. (2007). The impact of ecosystem connectivity on coral reef resilience. *Journal of Applied Ecology*, *45*(3), 854–862. https://doi.org/10.1111/j.1365-2664.2008.01459.x

Murphy, S. J., Hurlburt, H. E., & O'Brien, J. J. (1999). The connectivity of eddy variability in the Caribbean Sea, the Gulf of Mexico, and the Atlantic Ocean. *Journal of Geophysical Research: Oceans*, *104*(C1), 1431–1453. https://doi.org/10.1029/1998JC900010

Mutlu, E., Çinar, M. E., & Ergev, M. B. (2010). Distribution of soft-bottom polychaetes of the Levantine coast of Turkey, eastern Mediterranean Sea. *Journal of Marine Systems*, *79*(1–2), 23–35. http://www.sciencedirect.com/science/article/B6VF5-4WKK1JF-1/2/fadfa45e5f53b60018ed2ddf6b243895

Nekola, J. C., & McGill, B. J. (2014). Scale dependency in the functional form of the distance decay relationship. *Ecography*, *37*(4), 309–320. https://doi.org/https://doi.org/10.1111/j.1600-0587.2013.00407.x

Nekola, J. C., & White, P. S. (1999). The distance decay of similarity in biogeography and ecology. *Journal of Biogeography*, *26*, 867–878.

Oksanen, J., Guillaume Blanchet, G., Friendly, M., Kindt, R., Legendre, P., McGlinn, D., Minchin, P. R., O'Hara, R. B., Simpson, G. L., Solymos, P., Stevens, M. H. H., Szoecs, E., & Wagner, H. (2020). *vegan: Community Ecology Package* (R Package version 2.5.7). https://cran.r-project.org/package=vegan

Olsgard, F., & Somerfield, P. J. (2000). Surrogates in marine benthic investigations - which taxonimic unit to target? *Journal of Aquatic Ecosystem Stress and Recovery*, *7*, 25–42.

Ortiz-Lozano, L., Pérez-España, H., Granados-Barba, A., González-Gándara, C., Gutiérrez-Velázquez, A., & Martos, J. (2013). The Reef Corridor of the Southwest Gulf of Mexico: Challenges for its management and conservation. *Ocean & Coastal Management*, *86*, 22–32. https://doi.org/10.1016/j.ocecoaman.2013.10.006

Ortiz-Zamora, G., Huerta-Díaz, M. A., Salas-de-León, D. A., & Monreal-Gómez, M. A. (2002). Degrees of pyritization in the Gulf of Mexico in sediments influenced by the Coatzacoalcos and the Grijalva-Usumacinta rivers. *Ciencias Marinas*, *28*, 369–379.

Oug, E., Bakken, T., Kongsrud, J. A., & Alvestad, T. (2017). Polychaetous annelids in the deep Nordic Seas: Strong bathymetric gradients, low diversity and underdeveloped taxonomy. *Deep Sea Research Part II: Topical Studies in Oceanography*, *137*, 102–112. https://doi.org/https://doi.org/10.1016/j.dsr2.2016.06.016

Pamungkas, J., Glasby, C. J., Read, G. B., Wilson, S. P., & Costello, M. J. (2019). Progress and perspectives in the discovery of polychaete worms (Annelida) of the world. *Helgoland Marine Research*, *73*(1), 4. https://doi.org/10.1186/s10152-019-0524-z

Peres-Neto, P. R., Legendre, P., Dray, S., & Borcard, D. (2006). Variation Partitioning of Species Data Matrices: Estimation and Comparison of Fractions. *Ecology*, *87*(10), 2614–2625. https://doi.org/https://doi.org/10.1890/0012-9658(2006)87[2614:VPOSDM]2.0.CO;2

Probert, P. K., Read, G. B., Grove, S. L., & Rowden, A. A. (2001). Macrobenthic polychaete assemblages of the continental shelf and upper slope off the west coast of the South Island, New Zealand. *New Zealand Journal of Marine and Freshwater Research*, *35*(5), 971–984. https://doi.org/10.1080/00288330.2001.9517057

Quiroz-Martínez, B., Hernández-Alcántara, P., Salas-de León, D. A., Solís-Weiss, V., & Monreal-Gómez, M. A. (2022). Depth-range distribution and diversity patterns of a polychaete (Annelida) community in the continental shelf of the Southern Gulf of Mexico. *Estuarine, Coastal and Shelf Science*, *265*. https://doi.org/https://doi.org/10.1016/j.ecss.2021.107739

Quiroz-Martínez, B., Hernández-Alcántara, P., Salas-de-León, D. A., & Solís-Weiss, V. (2021). Polychaete (Annelida) Diversity Patterns in Southern Gulf of Mexico: The Influence of Spatial Structure and Environmental Variables. In *Diversity* (Vol. 13, Issue 9). https://doi.org/10.3390/d13090425

Quiroz-Martínez, B., Schmitt, F. G., & Dauvin, J. C. (2012). Statistical analysis of polychaete population density: dynamics of dominant species and scaling properties in relative abundance fluctuations. *Nonlinear Processes in Geophysics*, *9*, 45–52. https://doi.org/https://doi.org/10.5194/npg-19-45-2012

R Development Core Team, R. (2020). R: A Language and Environment for Statistical Computing. In R. D. C. Team (Ed.), *R Foundation for Statistical Computing*. R Foundation for Statistical Computing. https://doi.org/10.1007/978-3-540-74686-7

Read, G., & Fauchald, K. (2021). *World Polychaeta Database.*

Salas-de-León, D. A., Monreal-Gómez, M. A., Díaz-Flores, M. A., Salas-Monreal, D., Velasco-Mendoza, H., Riverón-Enzástiga, M. L., & Ortiz-Zamora, G. (2008). Role of Near-Bottom Currents in the Distribution of Sediments within the Southern Bay of

Campeche, Gulf of Mexico. *Journal of Coastal Research*, *24*(6), 1487–1494. http://www.jstor.org/stable/40065132

Salas-Monreal, D., Marin-Hernandez, M., Salas-Perez, J. de J., Salas-de-Leon, D. A., Monreal-Gomez, M. A., & Perez-España, H. (2018). Coral reef connectivity within the Western Gulf of Mexico. *Journal of Marine Systems*, *179*, 88–99. https://doi.org/https://doi.org/10.1016/j.jmarsys.2017.12.001

Santibañez-Aguascalientes, N. A., Borja, Á., & Ardisson, P.-L. (2021). Sustainability situations for the southern Gulf of Mexico seafloor, based on environmental, benthic, and socioeconomic indicators. *Science of The Total Environment*, *787*, 147726. https://doi.org/https://doi.org/10.1016/j.scitotenv.2021.147726

Santibañez-Aguascalientes, N. A., Borja, Á., & Ardisson, P.-L. (2023). Assessing the large-scale and long-term changes in the southern Gulf of Mexico benthic ecological status under natural and human-induced disturbances. *Estuarine, Coastal and Shelf Science*, *283*, 108282. https://doi.org/https://doi.org/10.1016/j.ecss.2023.108282

Santibañez-Aguascalientes, N. A., Borja, Á., Kuk-Dzul, J. G., Montero-Muñoz, J. L., & Ardisson, P.-L. (2018). Assessing benthic ecological status under impoverished faunal situations: A case study from the southern Gulf of Mexico. *Ecological Indicators*, *91*, 679–688. https://doi.org/https://doi.org/10.1016/j.ecolind.2018.04.048

Santibañez-Aguascalientes, N. A., Borja, Á., Montero-Muñoz, J. L., Herrera-Dorantes, M. T., & Ardisson, P.-L. (2020). Setting reference conditions to assess the ecological status of the sublittoral and bathyal benthic communities of the southern Gulf of Mexico. *Ecological Indicators*, *111*, 105964. https://doi.org/https://doi.org/10.1016/j.ecolind.2019.105964

Sanvicente-Añorve, L., Zavala-Hidalgo, J., Allende-Arandía, M. E., & Hermoso-Salazar, M. (2014). Connectivity patterns among coral reef systems in the southern Gulf of Mexico. *Marine Ecology Progress Series*, *498*, 27–41. https://doi.org/https://doi.org/10.3354/meps10631

Schnurr, S., Osborn, K. J., Malyutina, M., Jennings, R., Brix, S., Driskell, A., Svavarsson, J., & Martinez Arbizu, P. (2018). Hidden diversity in two species complexes of munnopsid isopods (Crustacea) at the transition between the northernmost North Atlantic and the Nordic Seas. *Marine Biodiversity*, *48*(2), 813–843. https://doi.org/https://doi.org/10.1007/s12526-018-0877-6

Semprucci, F., Facca, C., Ferrigno, F., Balsamo, M., Sfriso, A., & Sandulli, R. (2019). Biotic and abiotic factors affecting seasonal and spatial distribution of meiofauna and macrophytobenthos in transitional coastal waters. *Estuarine, Coastal and Shelf Science*, *219*, 328–340. https://doi.org/https://doi.org/10.1016/j.ecss.2019.02.008

Snelgrove, P. V. R., & Butman, C. A. (1994). Animal-sediment relationships revisited: cause versus effect. *Oceanography and Marine Biology: An Annual Review.*, *32*, 111–177.

Solís-Weiss, V., Hernández-Alcántara, P., Granados-Barba, A., Ochoa-Rivera, A., & Palomar-Morales, I. (1995). *Atlas de Anélidos Poliquetos de la Plataforma Continental del sur del Golfo de México* (Issue Proyecto PO/52). CONABIO.

Soto, L. A., & Escobar-Briones, E. (1995). Coupling mechanisms related to benthic production in the SW Gulf of Mexico. *Biology and Ecology of Shallow Coastal Waters. Proc. 28th European Marine Biology Symposium, Iraklio, Crete, 1993*, 233–242. https://www.scopus.com/inward/record.uri?eid=2-s2.0-0029548631&partnerID=40&md5=7c20451ad1c2b7b3a14d46f0adc1899a

Thrush, S. F., Hewitt, J. E., Cummings, V. J., Norkko, A., & Chiantore, M. (2010). β-Diversity and Species Accumulation in Antarctic Coastal Benthos: Influence of Habitat, Distance and Productivity on Ecological Connectivity. *PLOS ONE*, *5*(7), e11899. https://doi.org/10.1371/journal.pone.0011899

Treml, E. A., Ford, J. R., Black, K. P., & Swearer, S. E. (2015). Identifying the key biophysical drivers, connectivity outcomes, and metapopulation consequences of larval dispersal in the sea. *Movement Ecology*, *3*(1), 17. https://doi.org/10.1186/s40462-015-0045-6

Van Hoey, G., Degraer, S., & Vincx, M. (2004). Macrobenthic community structure of soft-bottom sediments at the Belgian Continental Shelf. *Estuarine, Coastal and Shelf Science*, *59*, 599–613.

Warwick, R. M. (1984). Species Size Distributions in Marine Benthic Communities. *Oecologia*, *61*(1), 32–41. http://www.jstor.org/stable/4217200

Warwick, R. M., & Clarke, A. (2001). Practical measures of marine biodiversity based on relatedness of species. *Oceanography and Marine Biology Annual Review*, *39*, 207–231.

Yáñez-Arancibia, A., & Sánchez-Gil, P. (1983). Environment behavior of Campeche Sound ecological system, off Terminos Lagoon, Mexico; preliminary results. *Anales Del Instituto de Ciencias Del Mar y Limnología UNAM*, *10*(1), 117–136.

1. Thank you for submitting the above manuscript to PLOS ONE. During our internal evaluation of the manuscript, we found that this manuscript is very closely related to your previous published article:

https://www.mdpi.com/1424-2818/13/9/425

Response

Our work attempts to describe the species richness patterns for this group, using multivariate analysis such as Cluster, Distance-based Redundancy Analysis, and Variation partitioning. We also analyzed the distance decay in similarity between sampling sites to measure beta diversity. Also, we define the key parameters that control the diversity in the study area. Although this manuscript relates to our previously published article (https://www.mdpi.com/1424-2818/13/9/425), it is substantially different. For one, we used the average taxonomic distinctness (Δ^+, a measure of the average degree to which species are related to each other) as a diversity measure in the present manuscript; this diversity index differs from the more classical Sorensen index because it includes taxonomic relationships between the species in an assemblage and it has been shown that indices based on the taxonomic relatedness of species may give additional information to other univariate measures of biodiversity. In addition, the clustering method used in this study was also different, as we used Ward's Clustering instead of the UPGMA clustering method. The new diversity index and clustering method combined allowed us to characterize the polychaete fauna from the Southern Gulf of Mexico in more detail, as shown by the three faunal assemblages described in our study instead of the two assemblages described in our previous study. Finally, the spatial variables were simulated by the analysis of the Distance-based Moran's Eigenvector maps (dbMEMs) to reveal the influence of spatial variables on the community structure of annelid polychaetes from the Gulf of Mexico. An analysis which was not performed on the previous article. All the analysis therefore provided different, complementary information to the one obtained on our previous manuscript and allowed us to refine the patterns of distribution of the polychaetes.

Formatted: Font: (Default) Times New Roman
