# Supplementary material for: Taxonomic distinctness and diversity patterns of a polychaete (Annelida) community on the continental shelf of the Southern Gulf of Mexico
Source: PLoS One. 2024 May 8;19(5):e0303250. doi: 10.1371/journal.pone.0303250 (PMC11078371; doi:10.1371/journal.pone.0303250)
Supplement: Attachment — Submitted filename: Response to Editor.docx [file pone.0303250.s001.docx]

1. Thank you for submitting the above manuscript to PLOS ONE. During our internal evaluation of the manuscript, we found that this manuscript is very closely related to your previous published article:

<https://www.mdpi.com/1424-2818/13/9/425>

Response

Our work attempts to describe the species richness patterns for this group, using multivariate analysis such as Cluster, Distance-based Redundancy Analysis, and Variation partitioning. We also analyzed the distance decay in similarity between sampling sites to measure beta diversity. Also, we define the key parameters that control the diversity in the study area. Although this manuscript relates to our previously published article (<https://www.mdpi.com/1424-2818/13/9/425>), it is substantially different. For one, we used the average taxonomic distinctness (Δ^+^, a measure of the average degree to which species are related to each other) as a diversity measure in the present manuscript; this diversity index differs from the more classical Sorensen index because it includes taxonomic relationships between the species in an assemblage and it has been shown that indices based on the taxonomic relatedness of species may give additional information to other univariate measures of biodiversity. In addition, the clustering method used in this study was also different, as we used Ward’s Clustering instead of the UPGMA clustering method. The new diversity index and clustering method combined allowed us to characterize the polychaete fauna from the Southern Gulf of Mexico in more detail, as shown by the three faunal assemblages described in our study instead of the two assemblages described in our previous study. Finally, the spatial variables were simulated by the analysis of the Distance-based Moran’s Eigenvector maps (dbMEMs) to reveal the influence of spatial variables on the community structure of annelid polychaetes from the Gulf of Mexico. An analysis which was not performed on the previous article. All the analysis therefore provided different, complementary information to the one obtained on our previous manuscript and allowed us to refine the patterns of distribution of the polychaetes.
