# Supplementary material for: Taxonomic distinctness and diversity patterns of a polychaete (Annelida) community on the continental shelf of the Southern Gulf of Mexico
Source: PLoS One. 2024 May 8;19(5):e0303250. doi: 10.1371/journal.pone.0303250 (PMC11078371; doi:10.1371/journal.pone.0303250)
Supplement: Attachment — Submitted filename: Quiroz Martinez manuscript.docx [file pone.0303250.s003.docx]

These are small changes in the puncttaion and wording in the text. I had no access to pdf files for some reason. Otherwise the paper was quite well written and the statistical analysis seems very well done. I did not check the bibliography to make sure they were all cited in the text, however. The figures are very good and adequately illustrate their results.

Line 33 Add the keyword **diversity**

Line 52 (Dray et al., 2012)**,** consequently discriminating

Line 54 puzzling factors thus statistical

Line 81 **O**n the continental shelf of the Gulf of Mexico the

Line 83 (Fauchald et al., 2009). However, most of these studies only

Line 84 fragmented or isolated way so information

Line 90 - 93 Gulf of Mexico. We assess the relative impact of spatial environmental components on polychaete community structure and examine the spatial relationships between polychaete assemblages and environmental variables

Line 103 extraction are constantly increasing

Line 104 port of Tampico in the

Line 105 State of Yucatan,

Line 108 Yucatan Peninsula’a coast and : the Bay of Campeche which is a narrow

Line 116-117 25% and: the “carbonate” region

Line 123 River system which modifies the temperature and salinity,

Line 124 frontogenetic processes **as well as** being

Line 130 southwest flow however, in the autumn and winter, the flow shifts

Line 188 sampling stgations. The

Line 191 Nekola & White, 1999) using the R package

Line 192 “boot.coefs”) as it adjust a

Line 375 In contrast, in other studies it has been found

Line 377 Snelgrove & Butman, 1994). Our results, founded

Line 378-379 (and the dbMEMs) was the primary factor, rather than depth, determining the polychaete

Line 381 carbonated bottoms where coarse sands

Line 383 eastern Campeche Bay syllid species richness

Line 402-403 complex process since a gradual transition between different assemblages frequently occurs along biological gradients

Line 436 Gulf of Mexico. In their view the geostrophic

Line 437-439 coral reef habitats with two open ocean pathways, in addition to the traditional coastal alongshore path, which is constrained to the continental shelf with the first linking

Line 440- 441 is driven in a south to southwestern pattern by the Caribbean current during the spring and summer, while during the autumn-winter seasons the flow reverts

Line 444 Veracruz reefs, connectivity
