# Supplementary material for: Taxonomic distinctness and diversity patterns of a polychaete (Annelida) community on the continental shelf of the Southern Gulf of Mexico
Source: PLoS One. 2024 May 8;19(5):e0303250. doi: 10.1371/journal.pone.0303250 (PMC11078371; doi:10.1371/journal.pone.0303250)
Supplement: Attachment — Submitted filename: Responses to the Journal Requirements.docx [file pone.0303250.s004.docx]

Responses to the Journal Requirements:

1. In this instance it seems there may be acceptable restrictions in place that prevent the public sharing of your minimal data. However, in line with our goal of ensuring long-term data availability to all interested researchers, PLOS’ Data Policy states that authors cannot be the sole named individuals responsible for ensuring data access (http://journals.plos.org/plosone/s/data-availability#loc-acceptable-data-sharing-methods).

Data requests to a non-author institutional point of contact, such as a data access or ethics committee, helps guarantee long term stability and availability of data. Providing interested researchers with a durable point of contact ensures data will be accessible even if an author changes email addresses, institutions, or becomes unavailable to answer requests.

Before we proceed with your manuscript, please also provide non-author contact information (phone/email/hyperlink) for a data access committee, ethics committee, or other institutional body to which data requests may be sent. If no institutional body is available to respond to requests for your minimal data, please consider if there any institutional representatives who did not collaborate in the study, and are not listed as authors on the manuscript, who would be able to hold the data and respond to external requests for data access? If so, please provide their contact information (i.e., email address). Please also provide details on how you will ensure persistent or long-term data storage and availability.

**Data are hosted by the SEANOE repository:**

**Quiroz-Martínez Benjamín, Hernández-Alcántara Pablo, Solís-Weiss Vivianne (2024). Dataset for taxonomic distinctness and diversity analysis of a polychaete species from the continental shelf of the Southern Gulf of Mexico. SEANOE.** [**https://doi.org/10.17882/98735**](https://doi.org/10.17882/98735)

2. We note that Figures 1 and 4 in your submission contain [map/satellite] images which may be copyrighted.

**Figures 1 and 4 were entirely done by the corresponding author using Matlab and Etopo data from NOAA (https://www.ncei.noaa.gov/products/etopo-global-relief-model) and were improved by Jorge Castro.**
