# Supplementary material for: Taxonomic distinctness and diversity patterns of a polychaete (Annelida) community on the continental shelf of the Southern Gulf of Mexico
Source: PLoS One. 2024 May 8;19(5):e0303250. doi: 10.1371/journal.pone.0303250 (PMC11078371; doi:10.1371/journal.pone.0303250)
Supplement: Attachment — Submitted filename: Response to reviewer 1.docx [file pone.0303250.s006.docx]

Response to Reviewer 1

Dear reviewer,

Dear reviewer, we are deeply grateful for the time and effort you have dedicated to reviewing our manuscript “Taxonomic distinctness and diversity patterns of a polychaete (Annelida) community in the continental shelf of the Southern Gulf of Mexico” for Plos One.

We thank you for your comments and suggestions, which improved the readability and flow of our manuscript. I have included all your edits and modifications in the tracked-changes reviewed manuscript.

Sincerely

Dr. Benjamín Quiroz-Martínez

Instituto de Ciencias del Mar y Limnología

Corresponding Author

Figure 3 was modified following the reviewers’ suggestion

Page 13 Discussion

Lines 292-293 In the Southern Gulf of Mexico, extensive extraction of oil and other hydrocarbons has occurred since the 1970s;

This was changed following the reviewers’ suggestion

Reviewed version

In the Southern Gulf of Mexico, extensive extraction of oil and other hydrocarbons has occurred since the 1970s;
